# All *Yersinia* Are Not Created Equal: Phenotypic Adaptation to Distinct Niches Within Mammalian Tissues

**DOI:** 10.3389/fcimb.2018.00261

**Published:** 2018-08-03

**Authors:** Kimberly M. Davis

**Affiliations:** W. Harry Feinstone Department of Molecular Microbiology and Immunology, Johns Hopkins Bloomberg School of Public Health, Baltimore, MD, United States

**Keywords:** *Yersinia* infections, inflammation, phagocytes, gene expression, heterogeneity

## Abstract

*Yersinia pseudotuberculosis* replicates within mammalian tissues to form clustered bacterial replication centers, called microcolonies. A subset of bacterial cells within microcolonies interact directly with host immune cells, and other subsets of bacteria only interact with other bacteria. This establishes a system where subsets of *Yersinia* have distinct gene expression profiles, which are driven by their unique microenvironments and cellular interactions. When this leads to alterations in virulence gene expression, small subsets of bacteria can play a critical role in supporting the replication of the bacterial population, and can drive the overall disease outcome. Based on the pathology of infections with each of the three *Yersinia* species that are pathogenic to humans, it is likely that this specialization of bacterial subsets occurs during all *Yersiniae* infections. This review will describe the pathology that occurs during infection with each of the three human pathogenic *Yersinia*, in terms of the structure of bacterial replication centers and the specific immune cell subsets that bacteria interact with, and will also describe the outcome these interactions have or may have on bacterial gene expression.

## Introduction

The genus *Yersinia* contains three species that are pathogenic to humans: *Yersinia pestis, Yersinia pseudotuberculosis*, and *Yersinia enterocolitica*. *Y. pseudotuberculosis* and *Y. enterocolitica* are intestinal pathogens that typically cause self-limiting gastroenteritis and mesenteric lymphadenitis (Hubbert et al., 1971; Paff et al., 1976). *Y. pestis* causes bubonic, septicemic, and pneumonic plague, and has acquired a distinct inoculation route over the course of its divergence from an ancestral *Y. pseudotuberculosis*, despite relatively few genetic changes (Achtman et al., 1999). *Y. pestis* is inoculated intradermally into a mammalian host through injection during a flea bite, and *Y. pestis* can also be directly inhaled to cause primary pneumonic plague. Despite distinctions in inoculation route, all three *Yersinia* species have a lymphotropism, meaning they quickly traffic to lymph nodes (LNs) or lymphoid tissues and preferentially colonize these tissues. All *Yersinia* can also spread systemically by accessing the bloodstream and colonizing deep tissue sites, such as the spleen and liver.

Several well-characterized virulence factors play an important role in the disease progression of *Yersinia* and the ability of the bacteria to survive and proliferate within multiple host tissues. The human pathogenic *Yersinia* all contain a virulence plasmid termed either pCD1 or pYV, which contains genes encoding a central virulence factor for *Yersinia:* the type-III secretion system (T3SS) and its associated effector proteins, Yops (Gemski et al., 1980; Portnoy et al., 1981). The *Yersinia* T3SS is a needle-like structure that injects Yops directly into the host cytosol. Yops collectively function to modulate host signaling pathways to either inhibit or promote inflammation, to inhibit phagocytosis and motility of host cells, and can also inhibit the production and release of antimicrobial substances, such as reactive oxygen species (ROS) (Schesser et al., 1998; Viboud and Bliska, 2005; Songsungthong et al., 2010, Zhang and Bliska, 2010.)

Despite many genetic similarities, there are several well-defined genetic differences between *Yersinia* species. *Y. pestis* has acquired two additional virulence plasmids and 32 additional chromosomal genes since its divergence from *Y. pseudotuberculosis*, but also lost the functionality of as many as 13% of *Y. pseudotuberculosis* gene products (Achtman et al., 1999; Parkhill et al., 2001; Deng et al., 2002; Chain et al., 2004). *Y. pestis* pseudogenes include adhesins, and the lack of these genes may impact the ability of the bacterium to adhere to host cells and inject the T3SS (Simonet et al., 1996; Chain et al., 2004). Multiple adhesins: invasin, Ail, YadA, plasminogen activator protease (Pla, expressed from one of the *Y. pestis*-specific virulence plasmids), and pH 6 antigen, have been shown to play a critical role in adherence to host cells and T3SS injection (Yang and Isberg, 1993; Marra and Isberg, 1997; Felek and Krukonis, 2009; Durand et al., 2010; Felek et al., 2010; Maldonado-Arocho et al., 2013). *Y. enterocolitica* and *Y. pseudotuberculosis* utilize invasin, Ail and YadA (Yang and Isberg, 1993; Marra and Isberg, 1997; Hudson and Bouton, 2006; Durand et al., 2010; Maldonado-Arocho et al., 2013; Paczosa et al., 2014; Mühlenkamp et al., 2015). *Y. pestis* has acquired inactivating mutations in both *yadA* and invasin, and instead uses Ail, Pla, and pH 6 antigen to promote host cell interactions (Bartra et al., 2008; Felek and Krukonis, 2009; Felek et al., 2010). Ail also plays a major role in promoting serum resistance and preventing complement deposition in all three *Yersinia*, which allows bacteria to survive within the bloodstream and resist neutrophil-mediated killing (Miller and Falkow, 1988; Bliska and Falkow, 1992; Pierson and Falkow, 1993; Kolodziejek et al., 2007; Bartra et al., 2008).

**Figure 1:**
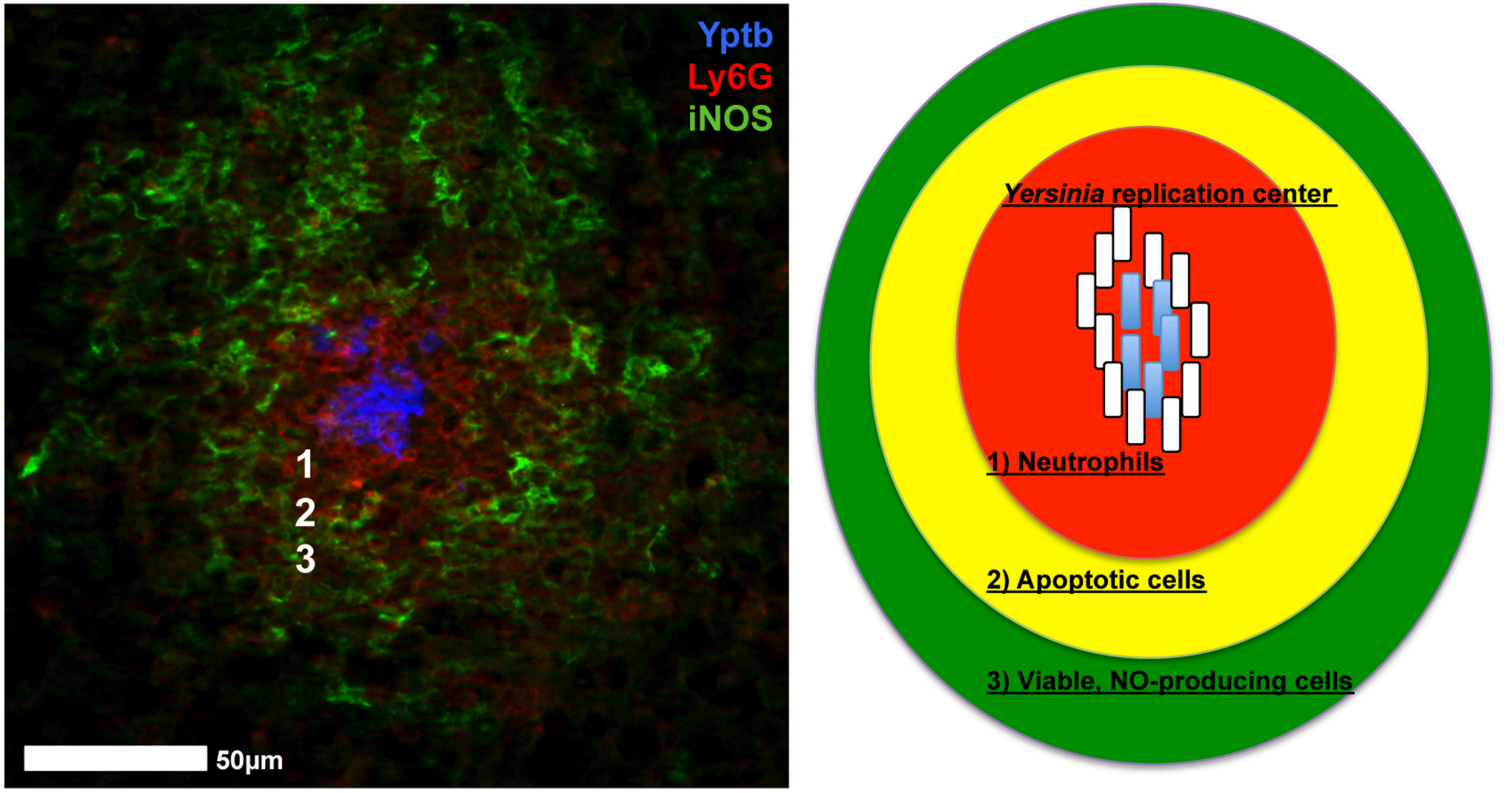
*Yersinia* replicate to form inflammatory lesions that contain several different types of immune cells. **Left**: Adapted from Davis et al. (2015). Neutrophils (Ly6G, red) and iNOS^+^ cells (iNOS, green) are recruited to replicating centers of *Y. pseudotuberculosis* (Yptb, blue). **Right**: Diagram of inflammatory lesions. (1) Neutrophils (red) form an inner layer surrounded by (2) Apoptotic cells (yellow; monocytes, some neutrophils). (3) Viable, NO producing cells (green; macrophages, dendritic cells, some neutrophils) form an outer layer of phagocytes. Peripheral bacterial cells (white rods) respond to host cell contact and diffusible antimicrobials, and allow interior bacteria (blue rods) to replicate. White numbers in the micrograph correspond with the numbered regions in the diagram.

*Yersinia* replicate extracellularly within host tissue sites to form clonal bacterial clusters (Simonet et al., 1990; Logsdon and Mecsas, 2006; Oellerich et al., 2007; Crimmins et al., 2012; Davis et al., 2015). The sites of *Y. pseudotuberculosis* replication have been termed lesions, microcolonies, and recently, pyogranulomas, but each term refers to the same structure, described in Figure 1 (Davis et al., 2015; Peterson et al., 2017; Zhang et al., 2018). This review will first describe disease progression in mammalian hosts, to introduce the different tissues *Yersinia* replicates within, and then discuss similarities and differences in the inflammatory lesions that form during *Yersinia* infection, with a focus on how interactions with distinct immune cell subsets may alter the gene expression profile of individual *Yersinia* cells within a replicating population.

## *Yersinia* human disease progression

### Enteropathogenic *Yersinia* disease progression

*Y. pseudotuberculosis* in an enteric pathogen that infects the terminal ileum of the small intestine and also colonizes associated lymphoid tissues, such as Peyer's patches and mesenteric lymph nodes (LNs) (Hubbert et al., 1971; Paff et al., 1976). In healthy individuals, robust neutrophil recruitment is typically sufficient to limit bacterial growth, and can result in clearance. However, in immunocompromised individuals, individuals with hemochromatosis (iron-overload disorder), or in a mouse model of infection, bacteria access the bloodstream and spread to deep tissue sites, such as the spleen and liver (Capron et al., 1981; Abbott et al., 1986; Barnes et al., 2006; Miller et al., 2016). Bacteria replicate within deep tissues to high numbers, which can cause a lethal infection in the mouse model. Replication within intestinal tissues also leads to spread to the cecum, where bacteria associate with cecal lymphoid follicles and can establish a long term, persistent infection in the mouse (Fahlgren et al., 2014; Avican et al., 2015).

Like *Y. pseudotuberculosis, Y. enterocolitica* is an intestinal pathogen that initially colonizes the intestinal lumen and associated lymphoid tissues, such as Peyer's patches and mesenteric LNs. *Y. enterocolitica* can also spread to deep tissues following replication at intestinal sites (Heesemann et al., 1993; Viboud and Bliska, 2005; Oellerich et al., 2007). The disease progression for enteric *Yersinia* appears to be very similar, however, *Y. enterocolitica* is a relatively common cause of gastroenteritis, whereas patients rarely present with *Y. pseudotuberculosis* infection. This may be because *Y. enterocolitica* commonly colonizes many different species of livestock and wild game animals, which could lead to increased exposure rates within human populations, or because *Y. enterocolitica* may elicit a heightened inflammatory response resulting in more severe disease (Dube et al., 2001; Chlebicz and Slizewska, 2018; Syczylo et al., 2018).

### *Y. pestis* disease progression

*Y. pestis* is inoculated into the dermis of mammalian hosts after replication within the midgut of the flea. Within the flea, bacteria replicate within dense aggregates that partially block the proventricular valve of the flea, thus promoting dissemination into the mammalian host during a flea bloodmeal (Hinnebusch et al., 2017). *Y. pestis* travels from the injection site rapidly into local draining LNs (dLNs), where colonization is established within the mammalian host. In mouse models of infection, bacteria have been shown to traffic to dLNs intracellularly within dendritic cells and monocytes, and also extracellularly within the lymphatic system (Shannon et al., 2013, 2015; St. John et al., 2014; Gonzalez et al., 2015). The exact route depends on the inoculation (intradermal or subcutaneous) and also the virulence of the bacterial strain. Following initial trafficking, bacteria appear to occupy an extracellular niche. Bacteria initially replicate within dLNs with limited inflammation, however as bacteria continue to replicate, high levels of inflammatory immune cell recruitment and tissue damage occurs. This forms the characteristic inflamed LNs, or buboes, that define bubonic plague (Butler, 1994). Tissue damage within LNs promotes bacterial bloodstream access, which initiates septicemic plague, and leads to colonization of deep tissues such as the spleen and liver (Perry and Fetherston, 1997). Septicemic plague can also occur immediately following flea-borne transmission, if the flea bite occurs deep within dermal tissues (Sebbane et al., 2006a). High levels of bacteria within the bloodstream can promote the development of a secondary pneumonic plague. Following bacterial replication in the lung, infected individuals can expel aerosols containing *Y. pestis*, which disseminate and cause primary pneumonic plague in new hosts. Septicemic plague also contributes to dissemination when a flea takes a bloodmeal from an infected host, which then restarts the *Y. pestis* life cycle in the flea vector (Perry and Fetherston, 1997; Hinnebusch et al., 2017).

### Host response to *Yersinia*, and *Yersinia* responses to the host

*Yersinia* encounter many different tissue sites during infection of mammalian hosts, which forces bacteria to adapt to multiple different environments within a single host organism. Recruited inflammatory cells attempt to kill *Yersinia* through phagocytosis or by releasing antimicrobial substances to eliminate extracellular bacteria. *Yersinia*, in turn, can inhibit phagocytosis and detoxify many of these antimicrobials. The genes required to battle the host response have been largely defined by transcriptional data and mutational analyses, that indicate a given gene or pathway contributes to virulence at the population level. Interestingly, distinct subsets of the bacterial population respond differently, depending on their interactions with, and proximity to, different host cell subsets (Marteyn et al., 2010; Burton et al., 2014; Davis et al., 2015). These studies have described host-driven heterogeneity during *Salmonella enterica* serovar Typhimurium and *Shigella flexineri* infections, in addition to *Yersinia*, which may indicate this is a widespread phenomenon within pathogenic bacterial populations. Although much is still unknown concerning *Yersinia* heterogeneity within different tissue sites, we will describe what has been shown thus far, and which pathways may also be utilized in other tissues based on similarities in host cell interactions.

### Lymph nodes

It has been hypothesized that the ability of *Y. pestis* to disseminate from dermal tissues and replicate within draining lymph nodes (dLNs) is due to the presence of Pla protease, which was acquired on an additional virulence plasmid after *Y. pestis* diverged from *Y. pseudotuberculosis* (Welkos et al., 1997; Lathem et al., 2007). However, *Y. pseudotuberculosis* can also effectively replicate within the dermis and traffic to dLNs following intradermal inoculation (Guinet et al., 2008, 2015). The major pathological difference during infection with the two species was the ability of the immune response to contain bacterial replication. *Y. pseudotuberculosis* elicits robust neutrophil recruitment that surrounds and contains bacteria, whereas *Y. pestis* exists as individual bacteria and reaches heightened bacterial loads (Guinet et al., 2008). *Y. pestis* has acquired the ability to produce LPS with poor stimulatory activity and also produces an anti-phagocytic capsule, both of which could limit neutrophil interactions (Montminy et al., 2006; Dudte et al., 2017). There were also many examples of *Y. pestis* clustered together within the same infected tissues, but it was unclear if clustering promotes bacterial growth or clearance by the host. In the absence of clustering, *Y. pseudotuberculosis* replication within mesenteric LNs causes a lethal infection in the mouse, suggesting clustering promotes host survival (Peterson et al., 2017). *Y. pseudotuberculosis* was contained by a layer of apoptotic monocytes (Figure 1), which may also contain *Y. pseudotuberculosis* within dLNs. These studies suggest that the formation of microcolony, or pyogranuloma, structure contains infection and protects the host, however this structure also allows bacteria to cooperate and promote their own growth (Davis et al., 2015).

*Y. pestis* replication within dLNs is characterized by high bacterial loads and high levels of tissue destruction. Replication of *Y. pestis* within dLNs initially induces very little host cytokine expression, which is actively inhibited by components of the virulence plasmid including the T3SS (Comer et al., 2010). At later timepoints, neutrophils and additional phagocytes are recruited to infected LNs, and IL-17 is produced (Comer et al., 2010). In response, *Y. pestis* increases expression of reactive nitrogen species (RNS) detoxifying genes and iron scavenging genes, indicating *Y. pestis* is being exposed to the antimicrobial diffusible gas, nitric oxide (NO), and has limited access to iron during replication within the dLN (Sebbane et al., 2006b). NO was produced by recruited neutrophils, and likely other phagocytes, within the rat bubo, and the detoxifying gene, *hmp*, played an important role in promoting *Y. pestis* virulence (Sebbane et al., 2006b). It is likely that bacteria in the closest proximity to NO-producing phagocytes may specifically respond to this stress, and detoxify NO to protect other members of the population (Davis et al., 2015), although this hasn't yet been investigated. It will also be interesting to determine if iron limitation is uniformly experienced across LNs, or if this is specific to a subset of cells. Interestingly there was little evidence for a bacterial response to reactive oxygen species (ROS) in dLNs, and only one superoxide dismutase increased in transcript levels. A mutant strain lacking this superoxide dismutase (*sodA*) was fully virulent, which indicates *Y. pestis* effectively inhibits this response within LNs (Sebbane et al., 2006b).

### Deep tissues

Multiple immune cell types are recruited to deep tissues during infection, and *Yersinia* effectively responds to create a hospitable environment for bacterial replication. Neutrophils directly interact with clustered centers of extracellular *Y. pseudotuberculosis*, and additional phagocytes are maintained at a distance relative to the bacterial centers (Figure 1). The layer of NO-producing cells likely contains a mixed population of monocytes, macrophages, and dendritic cells, and is adjacent to an apoptotic cell layer likely composed of monocytes (Davis et al., 2015; Peterson et al., 2017; Zhang et al., 2018). In the spleen, only the bacteria at the periphery of replicating centers respond to NO, and detoxify NO to prevent further diffusion into the microcolony, thus protecting interior bacteria (Davis et al., 2015). NO was detoxified by Hmp, which was expressed in response to the *Nos2* gene product, inducible nitric oxide synthase, indicating *hmp* expression was a specific response to NO. This example of cooperative behavior likely also exists within mesenteric LNs, based on the recruitment of NO-producing cells, and the similarities in these microcolony or pyogranuloma structures (Zhang et al., 2018). Peripheral *Y. pseudotuberculosis* cells also respond to neutrophil contact in the spleen by expressing heightened levels of the T3SS (Pettersson et al., 1996; Davis et al., 2015). It remains unclear if heightened expression signifies a T3SS translocation event, or if this is due to prolonged host cell contact. Heightened T3SS expression could occur in multiple tissue sites and response to many host cell types, as it is clear that *Yersinia* can inject T3SS effector proteins into many different immune cell types during infection (Durand et al., 2010).

*Y. pestis* and *Y. enterocolitica* actively inhibit immune cell recruitment to the spleen by limiting CCR2 signaling and promoting Gal-1 expression, respectively (Kerschen et al., 2004; Ye et al., 2009, 2011; Davicino et al., 2017). In the liver, inflammatory monocytes and dendritic cells are recruited to sites of *Y. pestis* infection, and YopM promotes cell death of neutrophils and macrophages leading to the formation of an apoptotic layer of host cells around replicating bacterial centers (Ye et al., 2014). This mirrors the layer of apoptotic cells observed within *Y. pseudotuberculosis-*infected mesenteric LNs (Figure 1) (Peterson et al., 2017). iNOS^+^ cells are also recruited to *Y. pestis* and *Y. enterocolitica* lesions, suggesting that subsets of peripheral cells may also express *hmp* to protect other individuals within the population. This also suggests that there may be quite a few similarities in the host response to *Yersinia* within deep tissues, unlike the pathological distinctions that are observed within infected LNs. This also indicates that all *Yersinia* may respond to similar stresses within deep tissues, and supports the idea that the cooperative behavior described for *Y. pseudotuberculosis* may also occur within other *Yersinia* infections, potentially even by expressing the same stress response pathways in distinct subsets of bacteria.

Neutrophils are a major producer of reactive oxygen species (ROS), but production can be inhibited by the T3SS, so it remains unclear if ROS restricts *Yersinia* growth in tissues. Mutant *Y. enterocolitica* lacking superoxide dismutase (*sodA*) were attenuated within the spleen and liver, but this could be due to sensitivity to endogeneous ROS generated during bacterial aerobic respiration, or host-derived ROS (Roggenkamp et al., 1997). Transcriptional responses to ROS have not been observed with *Y. pestis* or *Y. pseudotuberculosis*, and a *Y. pestis sodA* mutant was not attenuated for growth in buboes or systemic infection (Sebbane et al., 2006b; Davis et al., 2015). However, a ROS-sensitive *Y. pseudotuberculosis dusB-fis* mutant was attenuated in immunocompetent mice, and rescued in the absence of ROS production, suggesting ROS was produced during infection (Green et al., 2016). Fis is a nucleoid-associated protein that likely has pleiotropic effects on the bacterial response to ROS, which may explain why the *dusB-fis* mutant uncovered an impact of ROS on *Y. pseudotuberculosis* growth. WT *Y. pseudotuberculosis* growth was not impacted by the presence or absence of host-derived ROS, indicating the WT strain can effectively defend itself from ROS without significant transcriptional changes (Davis et al., 2015; Green et al., 2016).

### Intestinal tissue and peyer's patches

Following intestinal colonization, enteropathogenic *Yersinia* traverse M cells to form micrcolonies within Peyer's patches, which are specialized lymphoid tissues associated with the intestinal epithelium. The immune response to *Y. pseudotuberculosis* and *Y. enterocolitica* in Peyer's patches is characterized by neutrophil influx and a mixed T_H_17/T_H_1 response, in contrast to the T_H_17-specific response to *Y. pestis* within dLNs (Comer et al., 2010; Davicino et al., 2017; Nuss et al., 2017). *Y. pseudotuberculosis* responds by expressing genes to prevent phagocytosis and counteract iron deprivation and RNS, similar to *Y. pestis* within dLNs (Sebbane et al., 2006b; Nuss et al., 2017). *Y. enterocolitica* also actively inhibits neutrophil recruitment by a YopH-dependent mechanism that reduces CXCR2 surface expression on neutrophils (Dave et al., 2016). Microcolonies within the lamina propria contain CD4^+^ T cells and inflammatory macrophage and/or dendritic cell populations, in addition to CD8^+^ T cells. Interaction with CD8^+^ tissue-resident memory T cells within the lamina propria can effectively contain *Y. pseudotuberculosis* replication within the terminal ileum (Bergsbaken and Bevan, 2015).

Intestinal infection with sublethal doses of *Y. pseudotuberculosis* can establish a persistent, asymptomatic infection with sustained fecal shedding of bacteria, which may promote dissemination to additional hosts (Fahlgren et al., 2014; Avican et al., 2015). Persistence led to a dramatic shift in bacterial gene expression, which resembled growth at room temperature in bacteriological media, and was characterized by heightened expression of flagella and invasin, and downregulation of the T3SS (Avican et al., 2015; Heine et al., 2018). RovA was involved in these changes, and is known to positively regulate invasin and pH6 antigen expression. RovA expression is bistable in bacteriological media between 30 and 34°C, indicating bacteria utilize a bet-hedging approach to prepare a subset of the population to invade host cells, potentially as bacteria are moving through the host environment and the temperature is increasing (Quade et al., 2012; Nuss et al., 2016). RovA expression was also detected within a subset of the bacterial population replicating within the cecum, suggesting that RovA expression could be another example of cooperative behavior (Nuss et al., 2016).

### Lungs

Very little inflammation is observed early during primary pneumonic *Y. pestis* infection, similar to early timepoints within LNs during bubonic plague (Lathem et al., 2005; Guinet et al., 2008; Comer et al., 2010). However, by 48 h post-infection, inflammatory cells are localized around centers of replicating bacteria and bacteria are responding to the host environment (Lathem et al., 2005). Within the lung, *Y. pestis* upregulates expression of the T3SS, iron acquisition genes, and RNS detoxifying genes, including *hmp* (Lathem et al., 2005). In contrast, expression of pH 6 antigen, Pla, and ROS detoxifying genes were downregulated, indicating bacteria were not responding to ROS, and these adhesins was not required to replicate within the lungs (Lathem et al., 2005). Similar results were seen in a *Y. pseudotuberculosis* lung infection model, where Ail and YadA contribute to replication and dissemination to deep tissues, but pH 6 antigen was dispensible (Paczosa et al., 2014). It will be interesting to determine if heightened gene expression occurs within all the individual *Yersinia* cells replicating within the lung, or if distinct subsets of cells respond in different ways. Based on experiments with *Y. pseudotuberculosis*, individual *Y. pestis* cells may be expressing heightened levels of the T3SS or heightened levels of *hmp* depending on their spatial location relative to immune cell subsets.

### Concluding remarks

As *Yersinia* replicate within tissues, they form microcolonies where some individual cells interact directly with host immune cells, and other cells only interact with other bacteria. *Yersinia* species elicit similar inflammatory responses, characterized by recruitment of neutrophils, monocytes, macrophages, and dendritic cells, and all *Yersinia* appear to react similarly by upregulating expression of anti-phagocytic factors, RNS detoxifying genes, and iron scavenging genes. Several key questions remain: Are microcolonies protective for the host, or do they promote bacterial replication? Can we alter the host response to better contain bacterial growth? Limiting the host response impacts the containment of *Yersinia* within microcolonies and increases virulence, suggesting microcolonies protect the host (Peterson et al., 2017). However, disruption of microcolonies by attenuating *Yersinia* can promote clearance of bacteria, suggesting there is also a protective effect for bacteria (Davis et al., 2015). More future studies will be needed to determine if the host immune response can be manipulated to better contain and clear infection.

We are only beginning to understand how host cell interactions are driving differences in bacterial gene expression, and how small subsets of the bacterial population may be producing virulence genes that are critical for promoting disease. Better understanding and characterization of the heterogeneity in gene expression across bacterial populations, specifically in virulence gene expression, will have important implications for identifying potential drug targets for novel therapeutics. We believe the heterogeneity within *Yersinia* populations is likely also present within populations of other bacterial pathogens, and may be a widespread phenomenon that needs to be taken into account when designing antimicrobial therapeutics.

## Author contributions

The author confirms being the sole contributor of this work and approved it for publication.

### Conflict of interest statement

The author declares that the research was conducted in the absence of any commercial or financial relationships that could be construed as a potential conflict of interest.
